# Metagenomic Insights into the Effects of Fructooligosaccharides (FOS) on the Composition of Luminal and Mucosal Microbiota in C57BL/6J Mice, Especially the *Bifidobacterium* Composition

**DOI:** 10.3390/nu11102431

**Published:** 2019-10-12

**Authors:** Jiayu Gu, Bingyong Mao, Shumao Cui, Xuemei Liu, Hao Zhang, Jianxin Zhao, Wei Chen

**Affiliations:** 1 State Key Laboratory of Food Science and Technology, Jiangnan University, Wuxi 214122, China; gujiayu2018@126.com (J.G.); cuishumao@jiangnan.edu.cn (S.C.); 15766503836@163.com (X.L.); zhanghao61@jiangnan.edu.cn (H.Z.); zhaojianxin@jiangnan.edu.cn (J.Z.); chenwei66@jiangnan.edu.cn (W.C.); 2 School of Food Science and Technology, Jiangnan University, Wuxi 214122, China; 3 National Engineering Research Center for Functional Food, Jiangnan University, Wuxi 214122, China; 4 Beijing Innovation Center of Food Nutrition and Human Health, Beijing Technology and Business University (BTBU), Beijing 100048, China

**Keywords:** fructooligosaccharides, 16S rRNA metagenomic sequencing, *groEL*, luminal and mucosal microbiota, *Bifidobacterium pseudolongum*

## Abstract

Fructooligosaccharides (FOS) are considered prebiotics and have been proven to selectively promote the growth of *Bifidobacterium* in the gut. This study aimed to clarify the effects of FOS intake on the composition of luminal and mucosal microbiota in mice. Briefly, mice were fed a 0% or 25% FOS (*w*/*w*)-supplemented diet for four weeks, and the composition of luminal and mucosal microbiota, especially the *Bifidobacterium,* was analyzed by sequencing the V3–V4 region of 16S rRNA and *groEL* gene, respectively. After FOS intervention, there were significant increases in the total and wall weights of the cecum and the amount of total short-chain fatty acids (SCFAs) in the cecal contents of the mice. At the phylum level, the results showed a significant increase in the relative abundance of Actinobacteria in the contents and mucosa from the cecum to the distal colon in the FOS group. Besides *Bifidobacterium*, a significant increase was observed in the relative abundance of *Coprococcus* in all samples at the genus level, which may be partially related to the increase in butyric acid levels in the luminal contents. Furthermore, *groEL* sequencing revealed that *Bifidobacterium pseudolongum* was almost the sole bifidobacterial species in the luminal contents (>98%) and mucosa (>89%). These results indicated that FOS can selectively promote *B. pseudolongum* proliferation in the intestine, either in the lumen or the mucosa from the cecum to the distal colon. Further studies are required to reveal the competitive advantage of *B. pseudolongum* over other FOS-metabolizing bacteria and the response mechanisms of *B. pseudolongum* to FOS.

## 1. Introduction

The human gastrointestinal tract is an open microbial ecosystem with trillions of bacteria [1,2], including the beneficial and harmful bacteria that together form the normal intestinal microflora. The cecum and colon are denser and more diverse bacterial habitats than the stomach and small intestine. The intestinal bacteria are mainly distributed in the intestinal lumen and in the mucus secreted by the epithelial cells (the mucosa) [3,4]. Because of the differences in physiological structures and the gradients in available nutrients along the small intestine and colon, the composition of the bacterial community changes longitudinally [5,6]. In addition, the luminal microbiota differ from the mucosal microbiota within the same segment [7]. Most studies on intestinal microbiota have focused on feces rather than luminal and mucosal samples because feces are readily available [8,9]. However, luminal and mucosal samples of the cecum and colon should be investigated to understand the distribution, composition, and gradual changes of gut microbiota along the gastrointestinal tract. 

In the cecum and colon, commensal bacteria produce short-chain fatty acids (SCFAs) from the carbohydrates that pass undigested from the upper gastrointestinal tract. Some undigested carbohydrates are considered prebiotics because they stimulate the growth of beneficial bacteria and exert physiological effects on the host [10,11]. Fructooligosaccharides (FOS) are considered prebiotics and have been proven to modulate the composition of intestinal microbiota by preferentially increasing the abundance of *Bifidobacterium* in vitro and in vivo [12,13,14]. It is well known that FOS selectively stimulate the growth of *Bifidobacterium* in the gut [7,12]. Notably, we performed high-throughput sequencing of the *groEL* gene in our previous study to investigate the composition of *Bifidobacterium* at the species level and found that FOS selectively stimulated the growth of *Bifidobacterium pseudolongum* in mice feces [15]. 

Although several other genera, including *Lactobacillus*, *Coprococcus*, and *Enterococcus*, are also capable of utilizing FOS [7,16,17], *Bifidobacterium* has been found to show a competitive advantage over other genera in the gut after FOS intervention. Few studies have investigated the response mechanism of *Bifidobacterium* to FOS in the gut. To understand the interactions between FOS and intestinal microbiota, it is necessary to investigate the composition of mucosal and luminal microbiota in the gut, especially that of *Bifidobacterium* at the species level. 

Therefore, in this study, we fed mice a FOS-supplemented diet for four weeks and analyzed the composition of luminal and mucosal microbiota, in particular *Bifidobacterium* composition in the cecum and colon using MiSeq sequencing to clarify the relationships between FOS and the composition and distribution of intestinal microbiota.

## 2. Materials and Methods 

### 2.1. Chemicals and Reagents

FOS (GFn, HPLC purity 95.93%) containing 1F-fructofuranosylnystose (2.88%), nistose (32.45%), and 1-kestose (60.60%) were purchased from Bao Lingbao Biotechnology Co., Ltd. (Shandong, China).

The DNA extraction, polymerase chain reaction (PCR) amplification, quantification and sequencing kits, including FastDNA Spin Kit for Soil, QIAquick Gel Extraction Kit, Quanta iTico PicoGreen dsDNA Assay Kit, KAPA Biosystems Library Quantification Kit, QubitTM dsDNA BR Assay Kit, TruSeq DNA LT Sample Preparation Kit and MiSeq Reagent Kit, were used as described in our previous study [15].

### 2.2. Animals and Sample Collection

C57BL/6J mice (7 weeks, male) were purchased from Shanghai Experimental Animal Center (Shanghai, China). The 14 mice were allocated to two groups of 7 mice each. All mice were housed in an independent ventilation system in the Laboratory Animal Center of Jiangnan University. A 12-h light/dark cycle was maintained and the ambient temperature was controlled at 22 °C. The mice were allowed ad libitum access to food and water. All of the experiments in this study were approved by the ethics committee of Jiangnan University (JN No. 20160927-20161105 (67)) and were performed in line with the EU guidelines for experimental animals (Directive 2010/63/EU).

After 1-week adaptation, the mice were fed a diet supplemented with 0% or 25% FOS (*w*/*w*) for 4 weeks (control and FOS groups, respectively). A FOS diet was prepared by adding FOS to the control diet, partially replacing the glucose component. The diet formulas are shown in Table S1. At the end of the experiment, the luminal contents and mucosa of the cecum and colon were collected and weighed [9]. The amount of SCFAs in the contents was determined, and the microbiota composition in all samples was analyzed using the MiSeq platform (Illumina, Inc., San Diego, CA, USA). All samples were stored at −80 °C until analysis.

### 2.3. Determination of the Weight of Cecum

The cecum and colon of the mice were collected and weighed together to obtain the total weight. The luminal contents were collected and weighed separately to obtain the content weight, and the remains were then weighed to obtain the wall weight. The colon was divided into three segments- the proximal, middle, and distal colon, depending on their distance from the cecum. 

### 2.4. Determination of the pH and SCFAs Level of the Intestinal Content

A certain amount of the cecal and colonic contents was mixed with deionized water to obtain a 15 g/mL solution, which was centrifuged at 13,000× *g* for 2 min. The pH of the supernatant was determined using InLab Ultra-Micro-ISM electrode (Mettler Toledo International Inc., Columbus, OH, USA).

The amount of SCFAs, including acetic acid, propionic acid, butyric acid, isobutyric acid, valeric acid, and isovaleric acid, was determined using the method described by Mao et al. [9]. Briefly, the contents were suspended in saturated NaCl solution and acidified by sulfuric acid. SCFAs were then extracted by diethyl ether. The concentrations of SCFAs were analyzed by gas chromatography-mass spectrometry (GC-MS) and calculated by external standard method. The concentration was expressed as μmol/g of contents and the content was computed as μmol.

### 2.5. DNA Extraction and PCR Amplification of the V3–V4 Region and the groEL Gene

Bacterial genomes were extracted from the luminal contents and mucosa following the manufacturer’s instructions. The V3–V4 region of the 16S rRNA and the *groEL* gene were amplified by PCR. The primers and PCR programs were as described in our previous study [15]. 

### 2.6. Quantification, Sequencing, and Bioinformatic Analysis

The PCR products were quantified using the QubitTM dsDNA BR Assay Kit following the manufacturer’s instructions. Libraries were constructed using the TruSeq DNA LT Sample Preparation Kit and sequenced on the MiSeq platform (600 cycles-PE). 

The sequence reads were processed and screened according to the standards described previously [18]. Bioinformatic analyses, including sequence screening, reads assembly, operational taxonomic unit (OTU) establishment, OTU taxonomy, principal coordinate analysis (PCoA) analysis, α-diversity and β-diversity analysis, were performed using the QIIME package as described in our previous study [15].The linear discriminant analysis effect size (LEfSe) analysis [19] was performed to identify the most biologically informative features differentiating any two groups.

### 2.7. Data Statistics and Analysis

Differences between three groups were evaluated using analysis of variance, and those between two groups were assessed using the *t*-test (SPSS 16.0). A *p* value of less than 0.05 was considered to indicate statistical significance. 

## 3. Results

### 3.1. Cecal Weights in Different Groups

Compared with the control mice, the FOS-supplemented mice showed significantly increased cecal wall weight (*p* < 0.05) (Table 1) and three times higher cecal content weight (*p* < 0.01) (Table 1). The cecum of the FOS-supplemented mice was also bigger than that of the control mice. 

### 3.2. pH and SCFAs Levels of the Cecal and Colonic Contents

The pH of the luminal contents from the cecum to the distal colon was greater than 7 in the control group, whereas that of the contents of cecum, proximal colon, middle colon, and distal colon was significantly lower (6.13, 5.93, 6.12, and 6.43, respectively) in the FOS group (*p* < 0.05) (Table 2). Notably, no significant difference was observed between the pH of the four parts of the intestine in the FOS group.

The decrease in the pH of luminal contents of the intestine may be attributable to the production of SCFAs [1], by colonic bacteria via the fermentation of FOS passed undigested from the upper gastrointestinal tract. GC-MS revealed that the total SCFAs content of the cecum significantly increased after FOS intervention (*p* < 0.01, Table 3), consistent with the pH decrease in the cecum and colon. Acetic acid was found to be the most abundant SCFAs, followed by isovaleric acid, propionic acid, butyric acid, isovaleric acid and valeric acid. The amounts of SCFAs were calculated from the concentrations of SCFAs (Table S2) and the weight of cecal contents. No significant difference was observed in the concentrations of various SCFAs in the cecum between the control and FOS groups (Table S2, *p* > 0.05).

### 3.3. Effects of FOS on the Composition of Luminal and Mucosal Microbiota in Cecum and Colon

**Figure 1:**
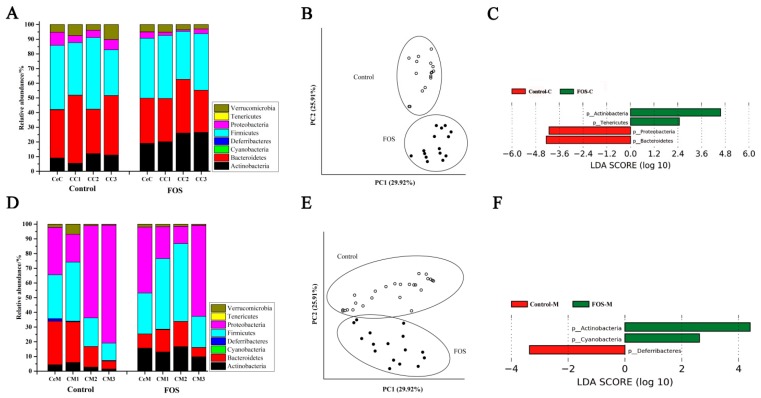
Changes in the composition of luminal (**A**) and mucosal (**D**) microbiota at the phylum level in the Control and FOS groups. Principal coordinate analysis (PCoA) score of luminal (**B**) and mucosal (**E**) microbiota based on the weighted UniFrac metrics. Linear discriminant analysis effect size (LEfSe) analysis of the differences between luminal (**C**) and mucosal (**F**) microbiota at the phylum level. CeC, CC1, CC2, and CC3 represent the contents of the cecum, proximal colon, middle colon and distal colon, respectively. CeM, CM1, CM2, and CM3 represent the mucosa of the cecum, proximal colon, middle colon and distal colon, respectively.

Bacteroidetes and Firmicutes were the most abundant phyla among the luminal microbiota of the cecum and colon, and the luminal microbiota showed similar composition from the cecum to the distal colon (Figure 1A). After FOS intervention, the relative abundance of Actinobacteria significantly increased in the luminal microbiota, whereas that of Bacteroidetes and Proteobacteria significantly reduced (Figure 1C). The PCoA results showed that the composition of luminal microbiota was well differentiated between the control and FOS group (Figure 1B). Among the mucosal microbiota, Bacteroidetes, Firmicutes, and Proteobacteria were the most abundant phyla, and the composition of mucosal microbiota varied from the cecum to the distal colon (Figure 1D). The relative abundance of Actinobacteria also significantly increased in the mucosal microbiota of the cecum and colon after FOS intervention (Figure 1F), indicating that FOS could increase the abundance of Actinobacteria in both the luminal contents and mucosa of the whole intestine.

**Figure 2:**
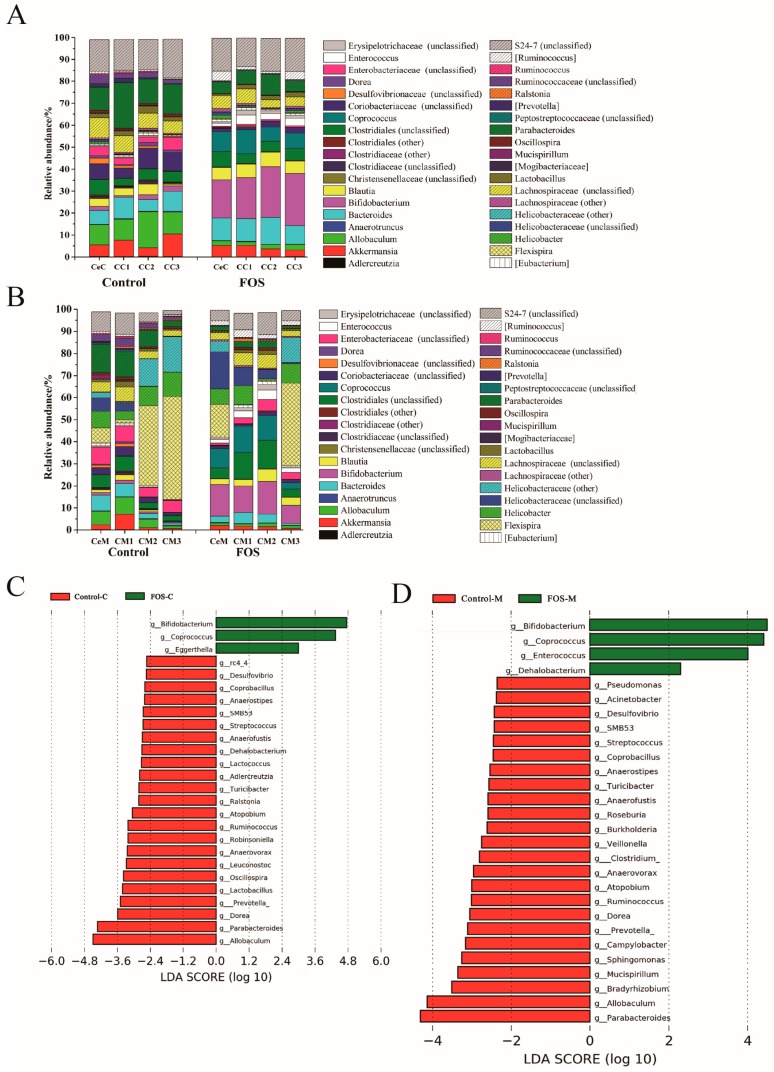
Changes in the composition of luminal (**A**) and mucosal (**B**) microbiota at the genus level in the control and FOS groups. LEfSe analysis of the differences between luminal (**C**) and mucosal (**D**) microbiota at the genus level. CeC, CC1, CC2 and CC3 reprsent the contents of the cecum, proximal colon, middle colon and distal colon, respectively. CeM, CM1, CM2 and CM3 represent the mucosa of the cecum, proximal colon, middle colon and distal colon, respectively.

In total, 288 genera were found in the luminal and mucosal samples, 38 of which showed a relative abundance of greater than 0.1% and accounted for >98% of the microbiota (Figure 2A,B). The data were analyzed using LEfSe to identify the key phylum and genus responsible for the differences in microbiota composition between the control and FOS groups. The abundances of 26 genera showed significant differences between the control and FOS groups (Figure 2C). Among the luminal microbiota, *Bifidobacterium* and *Coprococcus* showed significant increases in the relative abundances after FOS interventions, whereas *Allobaculum* and *Parabacteroides* showed significant reductions in their relative abundances (Figure 2C). The composition of mucosal microbiota varied from the cecum to the distal colon (Figure 2B), and also differed from that of the luminal microbiota (Figure S1). *Flexispira* and *Helicobacter* were the two major distinguishing genera between the luminal and mucosal microbiota (Figure S1); *Flexispira* accounted for 46.61% of the mucosal microbiota of the distal colon (Figure 2B). Consistent with the changes in luminal microbiota, mucosal microbiota showed significant increases in the relative abundances of *Bifidobacterium* and *Coprococcus* and significant decreases in the relative abundances of *Parabacteroides* and *Allobaculum* after FOS intervention (Figure 2D). 

**Figure 3:**
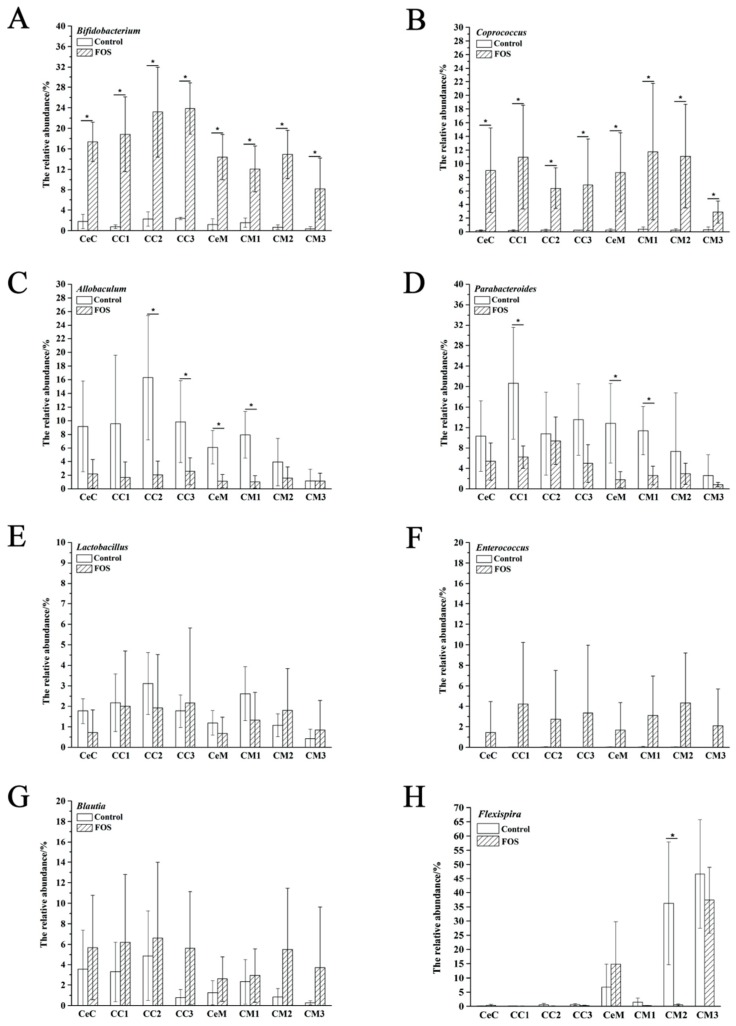
Changes in the relative abundance of specific genera, including *Bifidobacterium* (**A**), *Coprococcus* (**B**), *Allobaculum* (**C**), *Parabacteroides* (**D**), *Lactobacillus* (**E**), *Enterococcus* (**F**), *Blautia* (**G**), *Flexispira* (**H**). Significant differences (*p* < 0.05) are indicated with an asterisk (*).

The relative abundances of *Bifidobacterium* and *Coprococcus* were significantly increased from the cecum to the distal colon in both the luminal contents and mucosa (Figure 3A,B). However, the relative abundances of *Allobaculum* and *Parabacteroides* significantly reduced in the CC2, CC3, CeM, and CM1, (Figure 3C) and CC1, CeM, and CM1 (Figure 3D), respectively, and significant differences were found between the luminal and mucosal samples (Figure 2C,D). Reportedly, some *Lactobacillus* and *Enterococcus* species can metabolize FOS [7]. However, in our study, their relative abundances were not significantly increased in the luminal or mucosal samples after FOS intervention. The abundance of *Blautia* was previously reported to be significantly increased in mice feces after FOS intervention [15]. However, no significant increases were observed in its abundance in the luminal contents and mucosa from the cecum to the distal colon after FOS intervention in our study (Figure 3G), although its abundance varied greatly between mice. 

### 3.4. Effects of FOS on the Composition of Bifidobacterium in the Luminal Contents and Mucosa

**Figure 4:**
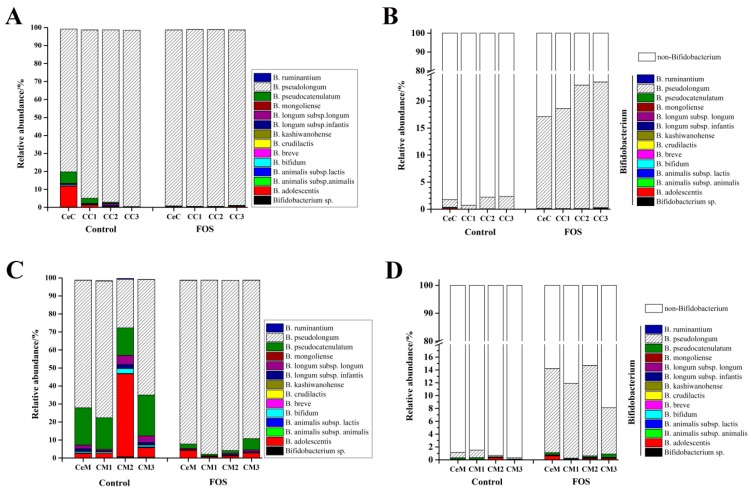
Effects of FOS on the relative abundance of *Bifidobacterium* species within the *Bifidobacterium* genus (**A**,**C**) and in the microbiota (**B**,**D**). A, and B indicate the contents and C, and D indicate the mucosa. “0” represents before FOS intervention, and “1” represents after FOS intervention.

14 species/subspecies in *Bifidobacterium* were detected in the contents and mucosa of the cecum and colon, and *B. pseudolongum* was the most abundant *Bifidobacterium* species (Figure 4A,B). The average relative abundance of *B. pseudolongum* was 91.7% and 59.3% in the luminal contents and mucosa, respectively. After FOS intervention, *B. pseudolongum* almost became the sole *Bifidobacterium* species with an average abundance of 98.0% in the luminal contents. As shown in Figure 4C, the composition of *Bifidobacterium* in the mucosa was different from that in the luminal contents. Following *B. pseudolongum*, *B. pseudocatenulatum* showed a high abundance of an average of 19.1% in the mucosa. The relative abundance of *B. adolescentis* was 45.9% in CM2. However, the relative abundance of *B. pseudolongum* reached an average of 92.5% throughout the large intestinal mucosa after FOS intervention. The relative abundance of *B. pseudolongum* reached an average of 20.4% and 11.5% in the luminal and mucosal microbiota, respectively. These results indicated that FOS significantly stimulated the growth of *B. pseudolongum* in both the luminal contents and mucosa from the cecum to the distal colon.

## 4. Discussion

Compared with the mice in the control group, significant enlargement of the cecum and softening of the luminal contents was observed in this study, which might be explained by the results showing that oligosaccharides supplementation increased the water content of the feces [20]. The total weight and wall weight of the cecum of mice in the FOS group were significantly higher than those of the control mice (Table 1), indicating that the cecal wall thickness did not decrease with the increase in the cecal size (data not shown) and this finding may be related to the production of SCFAs in the cecum [21]. The cecum is the main fermentation site for carbohydrates in mice, and the amounts of SCFAs, including acetic acid, propionic acid, butyric acid, isobutyric acid, valeric acid and isovaleric acid in the cecal contents significantly increased after FOS intervention, (Table 2). SCFAs provide energy for cell differentiation and proliferation, thereby increasing the crypt depth and cell density in the cecum [21], and consequently increasing the wall weight. Similar results for the changes in cecal weight were also obtained in rats fed different doses of lactose [22]. In addition to SCFAs, lactate and succinate might be produced after FOS fermentation. The gut permeability and inflammatory responses might be impacted due to stasis of the acidic environment [23].

The effects of FOS on the composition of the luminal and mucosal microbiota of mice were investigated via high-throughput MiSeq sequencing. Significant differences were observed in the composition of luminal and mucosal microbiota, consistent with our previous reports [9]. In addition, the composition of the luminal and mucosal microbiota also differed from that of the fecal microbiota. In most studies on intestinal microbiota, fecal samples are commonly used because of their easy availability. However, it is necessary to evaluate the luminal and mucosal samples to elucidate the changes in microbiota composition along the intestinal tract. FOS can only modulate the bacteria already existing in the gut; thus, FOS exerted different regulatory effects on the luminal and mucosal microbiota (Figure 2) depending on the differences between the original luminal and mucosal microbiota [9]. 

The LEfSe results indicated a significant increase in the relative abundance of Actinobacteria in both the luminal contents and mucosa from the cecum to the distal colon after FOS intervention (Figure 1), and *Bifidobacterium* contributed the most to this increase (Figure 2). This increase was consistent with the study of FOS ingestion in humans [24]. In addition, the relative abundance of *Coprococcus* was also significantly increased in the luminal and mucosal microbiota (Figure 3B), consistent with the previous results in feces [19]. *Coprococcus* is a genus of anaerobic cocci comprising three major species, of which *C. comes* and *C. eutactus* are capable of utilizing short-chain FOS [25]. As reported in the Bergey’s Manual of Systematic Bacteriology, the major products of carbohydrates fermentation by *Coprococcus* are butyric acid and acetic acid, followed by formic acid or propionic acid. Therefore, increases in the butyric acid content may be partially related to the increase in the abundance of *Coprococcus* [26]. 

Microbial distribution along the gastrointestinal tract is irregular and heterogeneous. As shown in Figure S1, large differences were found in the relative abundances of many genera between the luminal contents and mucosa, which may be related to the availability of various carbon sources. For example, the relative abundance of *Helicobacter* was higher in the mucosa than in the luminal contents, most likely because *Helicobacter* can use mucin as its carbon source [27], which is the main component of the intestinal mucosal barrier [28]. *Flexispira* was very similar to *Helicobacter* in taxonomy [29] and some members can cause gastrointestinal diseases and chronic diarrhea in animals [30]. After a four-week FOS intake, the relative abundance of *Flexispira* did not change significantly in the cecum (Figure 3H). In contrast, *Bifidobacterium* can metabolize several types of carbohydrates from diets [31], which allows it to survive extensively throughout the gut luminal contents and mucosa. No clear boundaries were observed for the bacteria distribution longitudinally along the intestine, although differences in the relative abundance were observed from the cecum to the distal colon. This finding is consistent with that of Welch et al. [32]. They established a model 15-member human gut microbiota in gnotobiotic mice and characterized the spatial distribution of the bacteria by in situ hybridization and spectral imaging analysis. They reported the luminal contents and mucosa of the proximal colon as an incompletely mixed bioreactor but not stratified compartments.

In our previous study, we identified 14 species in mice feces by high-throughput sequencing of the *groEL* gene, of which *B. pseudolongum* was the most abundant [15]. The *Bifidobacterium* composition of the luminal contents and mucosa was found to differ from that of feces. In addition to *B. pseudolongum*, *B. pseudocatenulatum* and *B. adolescentis* showed high relative abundances in the mucosa, and their relative abundances varied from cecum to the distal colon (Figure 4). FOS showed great effects on the composition of intestinal microbiota, especially of *Bifidobacterium*. After FOS intervention, *B. pseudolongum* became almost the sole *Bifidobacterium* species present in both the luminal contents and in the mucosa. Although *B. pseudocatenulatum* and *B. adolescentis* have been found to metabolize FOS in vitro [33], *B. pseudolongum* exerted a competitive advantage over the other *Bifidobacterium* species. Thus, the increase in the relative abundance of *Bifidobacterium* was almost entirely contributed by *B. pseudolongum* after FOS intervention after the short period of FOS intake. 

It has been reported that the microbiota may return to baseline levels after discontinuation of short-term prebiotics intake [24,34]. Mao et al. revealed that the levels of *Bifidobacterium* and *Olsenella* both notably increased after a FOS diet (25%) and the microbiota tended to revert to initial structure two weeks after FOS treatment ceased [18]. This study only reported the effects of short-term ingestion, and the long-term ‘functional’ effects of FOS should be studied. Therefore, further studies are required to determine microbial activities and bifidobacterial composition in mice that would compare short (four week), medium (eight weeks), and long (24–27 weeks) periods of FOS intake. 

The analysis of luminal and mucosal microbiota confirmed that FOS can selectively promote *B. pseudolongum* proliferation in the intestine, independent of the position of *B. pseudolongum* in the longitudinal and horizontal intestine. However, the mechanism of selective promotion remains unclear. Further studies should elucidate the competitive advantage of *B. pseudolongum* over other intestinal bacteria in the use of FOS and the reason for the proliferation allowed by the host gut. This would be helpful in clarifying the response mechanism of *B. pseudolongum* to FOS and the relationships between prebiotics and gut microbiota.

## Figures and Tables

**Table 1:** Cecal weights (g) in the control and fructooligosaccharides (FOS) groups.

Item	Control Group	FOS Group	*p* ^1^
Total weight	0.28 ± 0.04 ^b^	0.87 ± 0.16 ^a^	<0.01
Wall weight	0.10 ± 0.03 ^b^	0.26 ± 0.05 ^a^	<0.05
Content weight	0.18 ± 0.03 ^b^	0.61 ± 0.07 ^a^	<0.01

^1^ Significant differences between the control and FOS groups are indicated by different letters (a, b), and significance was accepted at *p* < 0.05.

**Table 2:** pH of the luminal contents of the cecum and proximal, middle, and distal colon in the control and FOS groups.

Item		pH		*p* ^1^
		Control Group	FOS Group	
Cecum		8.03 ± 0.12 ^a^	6.13 ± 0.06 ^b^	<0.001
Colon	Proximal	7.46 ± 0.15 ^a^	5.93 ± 0.66 ^b^	<0.05
	Middle	7.62 ± 0.26 ^a^	6.12 ± 0.29 ^b^	<0.001
	Distal	7.11 ± 0.42 ^a^	6.43 ± 0.26 ^b^	<0.05

^1^ Significant differences between the control and FOS groups are indicated by different letters (a, b), and significance was accepted at *p* < 0.05.

**Table 3:** Short-chain fatty acids (SCFAs) contents (μmol) of the cecum in the control and FOS groups (μmol) ^1^.

SCFAs	Control Group	FOS Group	*p* ^2^
Acetic acid	7.41 ± 2.60 ^b^	31.17 ± 11.08 ^a^	<0.001
Propionic acid	3.23 ± 0.81 ^b^	10.17 ± 4.71 ^a^	<0.01
Butyric acid	1.55 ± 0.36 ^b^	3.58 ± 0.88 ^a^	<0.01
Isobutyric acid	3.04 ± 0.73 ^b^	14.60 ± 7.15 ^a^	<0.01
Valeric acid	0.77 ± 0.23 ^b^	1.82 ± 0.44 ^a^	<0.01
Isovaleric acid	1.12 ± 0.28 ^b^	2.59 ± 0.64 ^a^	<0.01
Total	17.12 ± 4.10 ^b^	63.93 ± 15.74 ^a^	<0.01

^1^ The SCFA contents were calculated from the concentrations of SCFAs (Table S2) and the weight of cecal contents. ^2^ Significant differences between the control and FOS groups were indicated by different letters (a, b). Significance was accepted at *p* < 0.05.

## Supplementary Materials

The following supporting materials are available online at https://www.mdpi.com/2072-6643/11/10/2431/s1. Table S1: Dietary formula. Table S2: The SCFAs concentrations of the cecal content in the Control and FOS groups. Figure S1: LEfSe analysis on the differences of luminal and mucosal microbiota at the genus level in the Control group.
