# Supplementary material for: Metagenomic Insights into the Effects of Fructooligosaccharides (FOS) on the Composition of Luminal and Mucosal Microbiota in C57BL/6J Mice, Especially the Bifidobacterium Composition
Source: Nutrients. 2019 Oct 12;11(10):2431. doi: 10.3390/nu11102431 (PMC6835839; doi:10.3390/nu11102431)
Supplement: Supplementary file 1 [file nutrients-11-02431-s001.zip › nutrients-594881-supplementary.docx]

**Supplementary Materials**

**Table S1.** Dietary formula (g)

| **Formula** | **Control group** | **FOS group** |
| --- | --- | --- |
|  | 100g Dry matter |  |
| Casein hydrolysate | 19.7 | 19.7 |
| L-Cystine | 0.3 | 0.3 |
| Flour | 30 | 30 |
| Glucose | 34.75 | 9.75 |
| FOS | 0 | 25 |
| Cocoanut oil | 7.88 | 7.88 |
| soybean oil | 2.62 | 2.62 |
| Choline bitartrate | 0.25 | 0.25 |
| Vitamin mix | 1 | 1 |
| Mineral mix | 3.5 | 3.5 |

**Table S2.** The SCFAs concentrations of the cecal content in the Control and FOS groups (μmol/g).

| SCFAs | Control group | FOS group | *P*^1^ |
| --- | --- | --- | --- |
| Acetic acid | 40.88±17.99 | 44.05±9.13 | NS |
| Propionic acid | 17.21±2.24 | 14.02±2.77 | NS |
| Butyric acid | 8.30±0.99 ^a^ | 5.23±1.44 ^b^ | <0.05 |
| Isobutyric acid | 16.58±4.38 | 23.26±15.16 | NS |
| Valeric acid | 4.06±0.20 ^a^ | 2.64±0.64 ^b^ | <0.05 |
| Isovaleric acid | 5.96±0.49 ^a^ | 3.77±0.99 ^b^ | <0.05 |
| Total | 92.99±24.05 | 92.98±22.04 | NS |

^1^ Significant differences between the Control and FOS groups were indicated by different letters (a, b) and signiﬁcance was accepted at *P < 0.05*. NS = not significant.

Figure S1


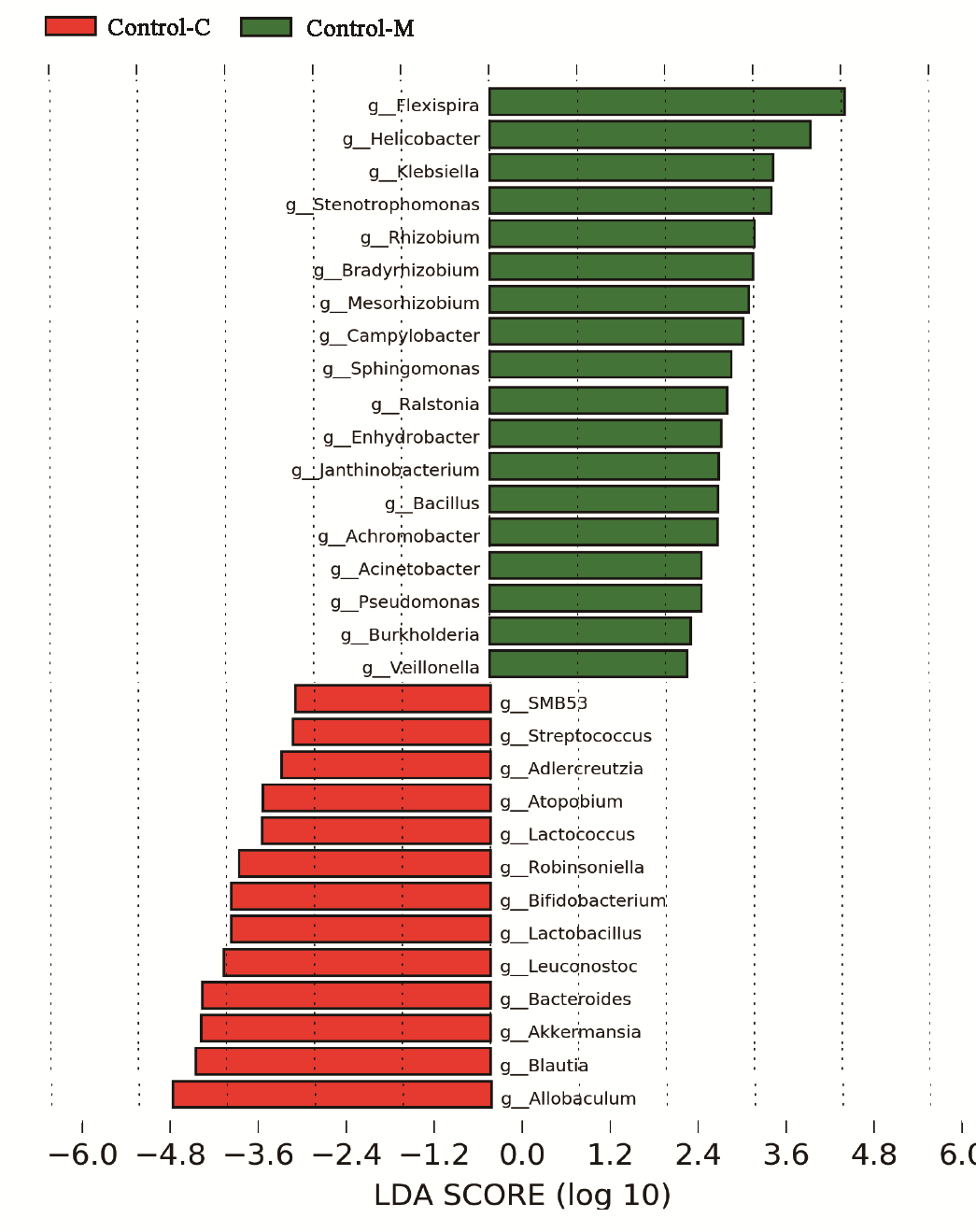


**Figure S1.** LEfSe analysis on the differences of luminal and mucosal microbiota in genus in the Control group.
